# Special Effect of Ionic Liquids on the Extraction of Flavonoid Glycosides from *Chrysanthemum morifolium* Ramat by Microwave Assistance

**DOI:** 10.3390/molecules20057683

**Published:** 2015-04-28

**Authors:** Ying Zhou, Datong Wu, Pengfei Cai, Guifang Cheng, Chaobiao Huang, Yuanjiang Pan

**Affiliations:** 1 College of Chemistry and Life Science, Zhejiang Normal University, Jinhua 321004, Zhejiang, China; E-Mails: zhouyingand@sina.cn (Y.Z.); 18757698316@163.com (G.C.); 2 Department of Chemistry, Zhejiang University, Hangzhou 310027, Zhejiang, China; E-Mails: wdt110328@zju.edu.cn (D.W.); caipeng2757@sina.com (P.C.); 3 Xingzhi College, Zhejiang Normal University, Jinhua 321004, Zhejiang, China

**Keywords:** ionic liquids, flavonoid glycosides, microwave-assisted extraction, *Chrysanthemum morifolium* Ramat

## Abstract

A microwave-assisted extraction approach based on ionic liquids of different chain lengths was successfully applied to the extraction of ten flavonoid glycosides from the flowering heads of *Chrysanthemum morifolium* Ramat. The pretreated sample was quantified by HPLC-ESI-MS^n^. The main components were identified as flavonoid glycosides, including three luteolin glycosides, three apigenin glycosides, three kaempferide glycosides, and one acacetin glycoside according to the characteristics of the corresponding CID mass spectrometric patterns. Eight ionic liquids from the imidazolium family with different chain lengths, namely, 1-alkyl-3-methylimidazolium bromide, [C_n_mim]Br, (n = 2–16) were studied as extraction medium in water. Results indicated that alkyl chain length had an irregular impact on the extraction efficiency. Moreover, the best extraction efficiency was achieved by 1-dodecyl-3-methylimidazolium bromide aqueous solution ([C_12_mim]Br). Besides the alkyl chain length of the cations, other factors influencing extraction efficiency were systematically investigated, including concentration of the IL solutions, extraction time, matrix-to-solvent ratio and irradiation power.

## 1. Introduction

Traditional Chinese Medicine (TCM), which is radically different from Western medicine, has formed a unique system to diagnose and cure diseases over its long history. As the cornerstone of formulas, herbal medicine is one of the major pillars of the Chinese medicine system. The Chinese Pharmacopoeia lists over 6,000 different medicinal substances in terms of their properties and the disharmonies they can cure. *Chrysanthemum morifolium* Ramat, namely, a flowering head from the Asteraceae family, which is widely popular in the South-East Asia region, has been used for a long time as a herbal medicine and herbal tea for the treatment or mitigation of headaches, allergies, inflammation, blood hypertension and eye-related diseases [1,2,3]. To study the effective activities of different phytochemicals and their functional mechanisms, several kinds of effective phytochemicals have been isolated or identified from this herbal medicine, such as organic acids [4,5], flavonoids [2,5,6], and polysaccharides [7,8]. Among them, significant amounts of flavonoid glycosides have been studied due to their diverse bioactivities [9,10,11,12,13], including radical-scavenging, anti-inflammatory, anti-cancer, and antioxidant properties,* etc.* Moreover, acacetin 7-*O*-galactoside and apigenin 7-*O*-β-d-(4''-caffeoyl) glucuronide isolated from *Chrysanthemum morifolium* Ramat exhibit satisfactory anti-HIV activity [14]. 

The sample pretreatment for the separation and enrichment of target bioactive compositions is one of the key steps in all research and development of herbal medicines in contemporary pharmaceuticals. Therefore, a variety of extraction methods have been applied to extracting the bioactive components from herbal botanicals [15,16,17,18,19,20,21,22]. Recently, in order to optimize the extraction conditions, several novel high efficiency and low cost extraction techniques have been developed aiming to increase the speed of extractions, such as microwave-assisted extraction (MAE) [17,18], ultrasonic-assisted extraction (UAE) [19,20],* etc.*

**Figure 1:**
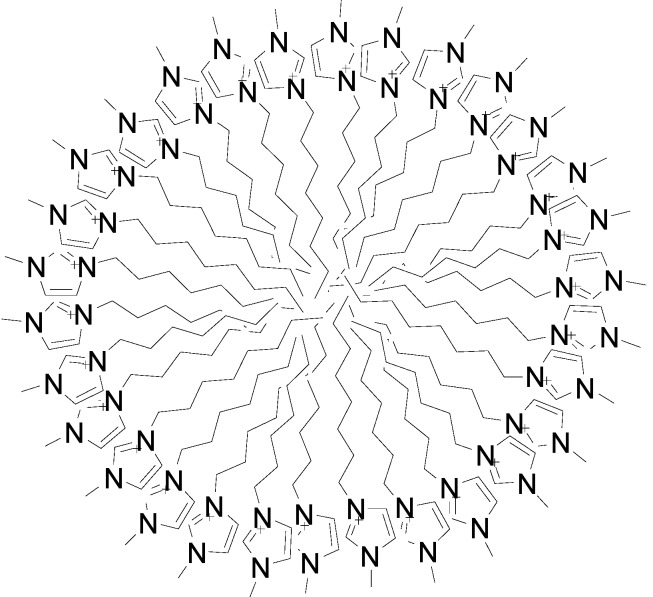
The aggregation morphology of dialklyimidazolium ILs in water.

Considering the advantages of low melting temperature, non-volatility, non-flammability and excellent thermal stability for recycle, ionic liquids (ILs) composed of organic cations and inorganic/organic anions have attracted much attention as green alternatives to traditional solvents. Although several former reports demonstrated that some ionic liquids have some certain toxicity [23,24,25,26,27], in recent years ionic liquids have still been applied in many scientific areas such as separation, chromatographic analysis and electrochemical analysis [28,29], especially for the extraction of value-added compounds [30]. Among them, the alkylimidazolium-type ILs, whose properties largely depend upon the alkyl chain lengths, have been applied to extracting bioactive phytochemicals [17,18,30,31,32,33,34,35,36] from various matrixes. If the alkyl chain lengths are long enough, ILs will act as surfactants that are able to self-aggregate into micelles when their concentration in an aqueous solution is above a specific value [37,38,39,40]. As shown in Figure 1, this kind of spherical micelle which has the alkyl chains inside and the imidazole groups outside, could increase the extraction surface area due to this morphological appearance. In addition, ILs can effectively absorb the microwave irradiation energy and transfer it to the matrixes, which helps the ILs form micellar aggregates more quickly. Many research groups have used ionic liquids with a microwave-assisted approach to extract or enrich the bioactive ingredients from herbal plants [41,42,43,44,45], but relatively few works have reported on how the chain length of cations affects the extraction efficiency when ILs are used as extraction medium.

In this study, aqueous solutions of eight ionic liquids which belong to the imidazolium family coupled with microwave assistance were applied to the specific extraction of flavonoid glycosides from the flowers of *Chrysanthemum morifolium* Ramat followed by HPLC-MS^n^ analysis. According to the characteristics of the CID mass spectrometric patterns, the extracted ten flavonoid glycosides were classified into four types bases on their aglycones, including three luteolin glycosides, three apigenin glycosides, three kaempferide glycosides and one acacetin glycoside. Due to the different sizes of the micelles formed by ILs [46], the extraction efficiencies change dramatically as the alkyl chain lengths of ILs increase. Besides the cation alkyl chain length, other factors influencing the extraction efficiency were systematically investigated, including extraction time, matrix-to-solvent ratio, concentration of ILs and irradiation power. 

## 2. Results and Discussion

### 2.1. Flavonoid Glycosides from Chrysanthemum morifolium Ramat

**Figure 2:**
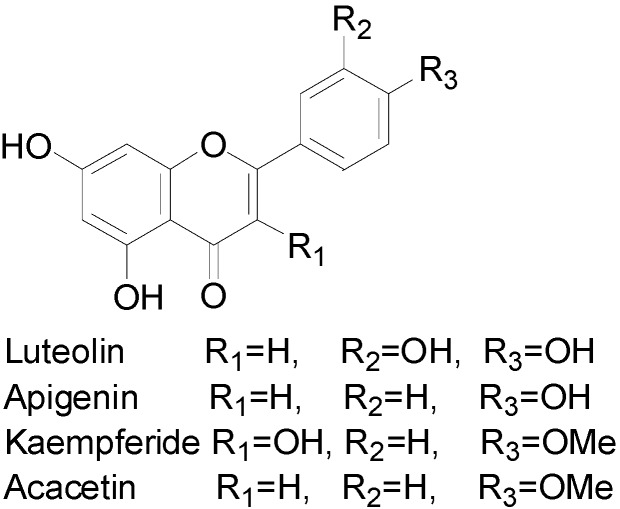
Four flavone aglycones in *Chrysanthemum morifolium* Ramat by HPLC-MS^n^ analysis.

**Figure 3:**
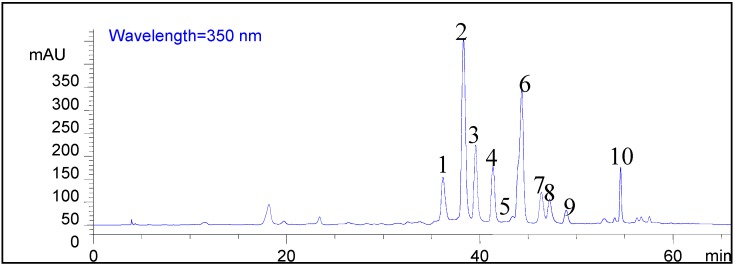
Ten flavonoid glycosides in *Chrysanthemum morifolium* Ramat by HPLC-MS^n^ analysis.

All of the products extracted from *Chrysanthemum morifolium* Ramat by every test throughout the whole series of experiments were all analyzed by HPLC, and the samples from the verification tests were analyzed by HPLC-MS^n^ (Figure 2). It was noteworthy that the formation of the diagnostic ions at *m/z* 151, 133 and 107 via a typical retro-Diels-Alder (RDA) reaction was the most significant cleavage behavior of flavonoids in the CID mass spectra [47]. The mass spectra indicated that four aglycone mass ionic fragments corresponding to luteolin (*m/z* 287/285), apigenin (*m/z* 271/269), kaempferide (*m/z* 301/-) and acacetin (*m/z* 285/283) appeared in positive/negative mode, respectively [48]. In principle, any of the hydroxyl groups on the flavonoids could be glycosylated by a O–C bond, although the 3- or 7-hydroxyls are favored. In addition, the existence of characteristic losses of 146, 162, 176 and 308 Da in the second-stage MS indicated that the glycosides were composed of rhamnose, glucose, glucuronic acid, rutinose and neohesperidoside, according to a previous report [49]. All ten flavonoid glycosides were assigned in the HPLC spectrum with the coded peaks depicted in Figure 3 and are conclusively identified in Table 1.

**Table 1:** HPLC results of extracts from *Chrysanthemum morifolium* Ramat.

NO.	t_R_	Mass(+/−)	MS^n^		Compound Name	Ref.
	min	Da	+	−		
1	36.185	595.0/593.4	448.8, 286.8	284.6	Luteolin 7-*O*-rutinoside	[5]
2	38.323	448.9/446.7	286.8	284.6	Luteolin 7-*O*-glucoside	[5]
3	39.578	462.9/462.3	286.8	284.7	Luteolin 7-*O*-glucuronide	[5]
4	41.374	579.0/579.0	432.8, 270.8	268.7	Apigenin 7-*O*-neohesperidoside	[5]
5	43.411	609.0/645.0	462.9, 300.9	608.2, 298.7	Kaempferide 7-*O*-neohesperidoside	[5]
6	44.347	432.9/430.7	270.8	268.7	Apigenin 7-*O*-glucoside	[5]
7	46.377	446.9/444.6	270.8	269.6, 174.6	Apigenin 7-*O*-glucuronide	[5]
8	47.226	462.9/−	300.8, 285.9	−	Kaempferide 7-*O*-glucoside	[5]
9	48.949	476.9/−	300.8, 285.8	−	Kaempferide 7-*O*-glucuronide	[5]
10	54.576	593.0/629.2	447.9, 285.8	590.7, 282.7, 267.8	Acacetin 7-*O*-rutinoside	[5]

### 2.2. Influence of Ionic Liquids

As mentioned above, at a suitable concentration, ILs will aggregate in water as organic media to extract target products [37,38,39,40]. However, their abilities to form micelles and the size of the micelles are significantly different, which is mainly due to their different alkyl chain lengths [50,51]. Therefore, in this study, eight different ionic liquids,* i.e.*, a series of 1-alkyl-3-methylimidazolium cations with different alkyl lengths from ethyl to hexadecyl were examined in aqueous solutions. In this test, the concentration, extraction time, matrix-to-solvent ratio and irradiation power were tentatively set as 0.25 M, 3 min, 0.5:10 g/mL and 500 W [16,17]. In order to quantify the extraction efficiency, luteolin 7-*O*-glucoside and apigenin 7-*O*-glucoside were selected as the indicator compounds. Additionally, different IL aqueous solutions (MAE) were prepared and contrasted with deionized water and ethanol (heat refluxing) as blanks to study the specific effects of the solvents.

**Figure 4:**
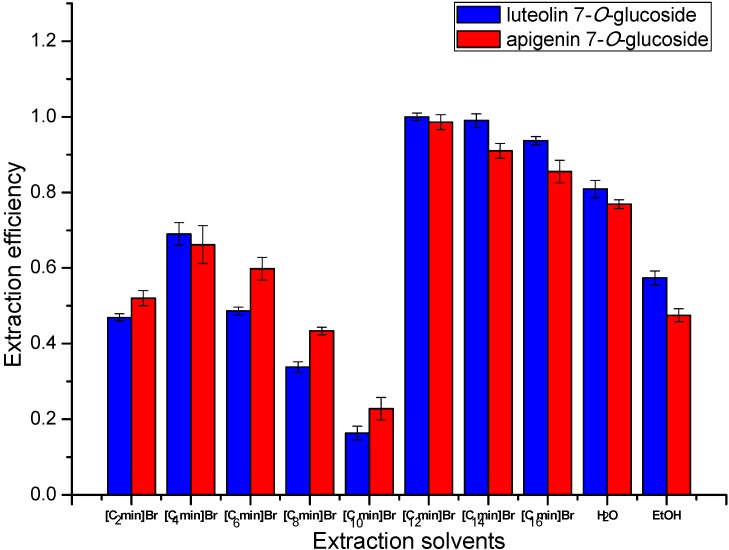
Extraction efficiency comparison of IL aqueous solutions with the blank control trial.

As shown in Figure 4, in aqueous solution, the alkyl chain length of the ILs had an irregular impact on the extraction efficiency of flavonoid glycosides. At the first half of this column chart, with the increase of the alkyl chain length, [C_4_mim]Br displayed the highest extraction efficiency. Then the extraction efficiency fell sharply, but this downward trend abruptly stopped at [C_10_mim]Br and suddenly [C_12_mim]Br showed the highest extraction efficiency of all the solvents. To our surprise, the extraction efficiency achieved by deionized water and ethanol (heat refluxing) was higher than that of some of the IL solutions. The results indicated that the function of the ionic liquids with various alkyl chain lengths was dramatically different. The ILs with long alkyl chains were able to act as amphiphilic molecules with lower critical micelle concentration (CMC, Table 2). On the other hand, the ionic liquids with short alkyl chains performed like salts causing strong ionic/charge-charge interactions that could reduce the compatibility with flavonoids and decrease the corresponding extraction efficiency. Therefore, the extraction efficiency achieved by the solutions with short alkyl-chain ILs was lower than those of deionized water and ethanol (heat refluxing). What was more, in the range of short alkyl lengths, the increasing alkyl length was not enough to make the IL act as a surfactant but improved the compatibility between the IL and flavonoid glycosides, and as a result, the [C_4_mim]Br solution presented a better result than [C_8_mim]Br solution. 

On the contrary, the alkyl length of [C_12_mim]Br was enough to make it show surfactant character and self-aggregate into micelles, which could improve the extraction surface area by possessing more interactions with the target compounds in aqueous solution by weak and non-covalent forces, such as hydrogen bonding, π-π, π-n, ionic/charge-charge, and dipole-dipole interactions. All these combined effects could lead to the apparent improvement of the extraction efficiency. As shown in the column chart, the efficiency of [C_12_mim]Br solution with microwave-assistance was much higher than that of ethanol (heat refluxing) and the solutions of ILs with short alkyl chains. However, when the alkyl chain lengths further increased, instead, the extraction efficiency started to decline again. This might due to the fact the unnecessarily length made the ILs aggregate in a more compact way which decreased the contact between the IL molecules and flavonoid glycosides in aqueous solution. Therefore, [C_12_mim]Br solution was selected as the optimum in the subsequent studies.

### 2.3. Optimization of the Extraction Process 

In addition to the alkyl chains of ILs, other variables including the concentration of aqueous solution, extraction time, matrix-to-solvent ratio and microwave power that may affect the extraction efficiency potentially were also taken into account. In this part, all the experiments were carried out with [C_12_mim]Br solution.

#### 2.3.1. Effect of Ionic Liquid Concentration for Extraction

**Figure 5:**
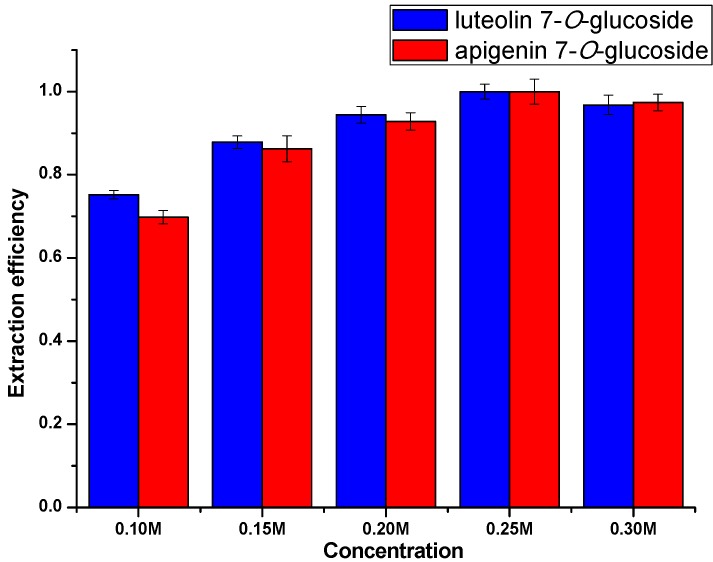
Extraction efficiency at different IL concentrations.

Previous research [52] showed that the concentration of ILs in aqueous solution would have a significant impact on the relative extraction efficiency. On this basis, the effects of different concentrations of [C_12_mim]Br aqueous solution (ranging from 0.1 M to 0.3 M) on the extraction of flavonoid glycosides from *Chrysanthemum morifolium* Ramat were studied. Other conditions including extraction time, matrix-to-solvent ratio and the irradiation power were tentatively set as 3 min, 0.5:10 g/mL and 500 W. As shown in Figure 5, the relative extraction efficiency gradually increased over the concentration range from 0.10 M to 0.25 M and declined after reaching the peak at 0.25 M, which resulted from the large amount of micelles being formed with the addition of IL. An IL-based surfactant could present a stronger interaction with flavonoid glycosides by hydrogen bonding and intermolecular forces, which could improve the relative extraction efficiency. On the other hand, the incresed extraction capacity couldn’t counteract the influence of increasing viscosity and diffusion resistance, which would make it more difficult for the IL solutions to penetrate into the herbal matrices. Thereby, the extraction efficiency decreased slightly. Finally, 0.25 M was selected as the optimized concentration in the following experiments.

#### 2.3.2. Effect of Extraction Time

**Figure 6:**
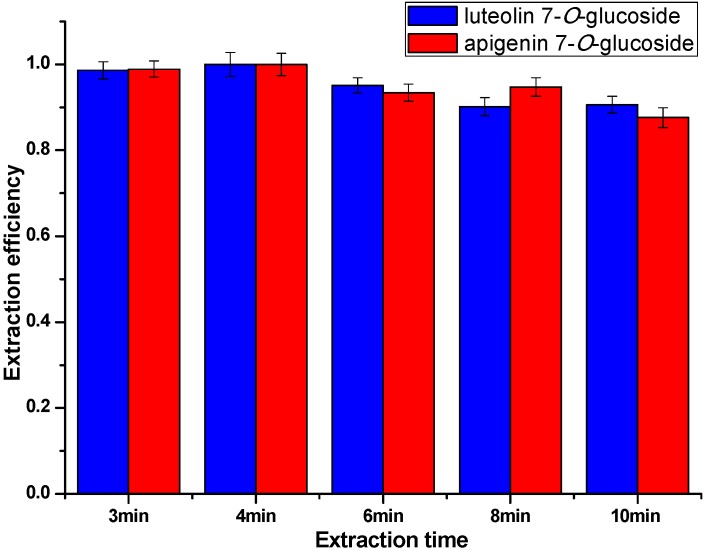
Extraction efficiency with different extraction times.

The optimization of extraction time was carried out with 3, 4, 6, 8 and 10 min extractions, respectively. Other conditions including matrix-to-solvent ratio and the irradiation power were tentatively set as 0.5:10 g/mL and 500 W. As shown in Figure 6, longer extraction duration didn’t result in a significant increase in extraction efficiency. Instead, after reaching the highest extraction efficiency at 4 min, the extraction efficiency gradually decreased. This might be attributed to the fact that the phase equilibrium of the system was achieved at 4 min, and after this point the water of the aqueous solution gradually evaporated as the experiment progressed, which could make the concentration of the solution improve slightly. Therefore, 4 min was selected as a suitable condition in the following studies. 

#### 2.3.3. Optimization of Extraction Matrix-to-Solvent Ratio 

**Figure 7:**
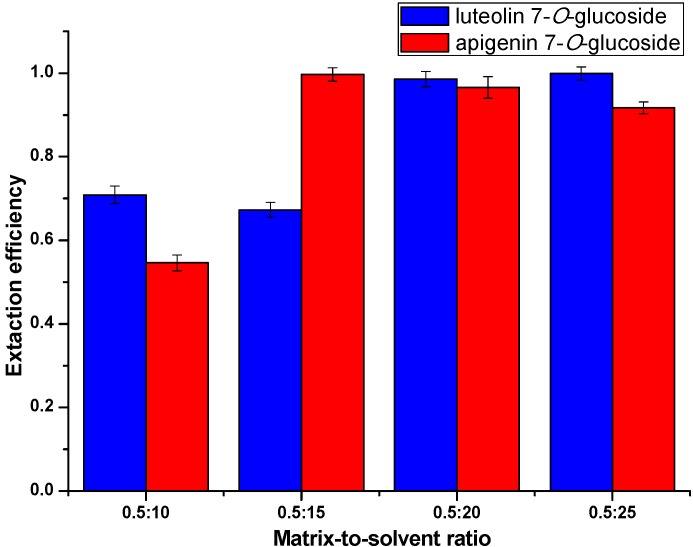
Extraction efficiency at different matrix-to-solvent ratios.

The matrix-to-solvent ratio (g/mL), which is related to the mass of *Chrysanthemum morifolium* Ramat and the volume of IL aqueous solution plays a key role in the extraction efficiency as well. Optimizing trials were carried out at different ratios of 0.5/10, 0.5/15, 0.5/20 and 0.5/25. Other conditions including concentration and extraction time were applied using the results optimized above, and the irradiation power was tentatively set as 500 W. The results (Figure 7) illustrate that the extraction efficiency was improved with the increase of matrix-to-solvent ratio and started leveling out at 0.5/20.

This might be because longer alkyl chains of the ILs result in a decrease of water miscibility and an increase of viscosity. Thus, to reach the maximum utilization of ILs, the matrix-to-solvent ratio was set at 0.5/20 g/mL.

#### 2.3.4. Effect of Extraction Power

**Figure 8:**
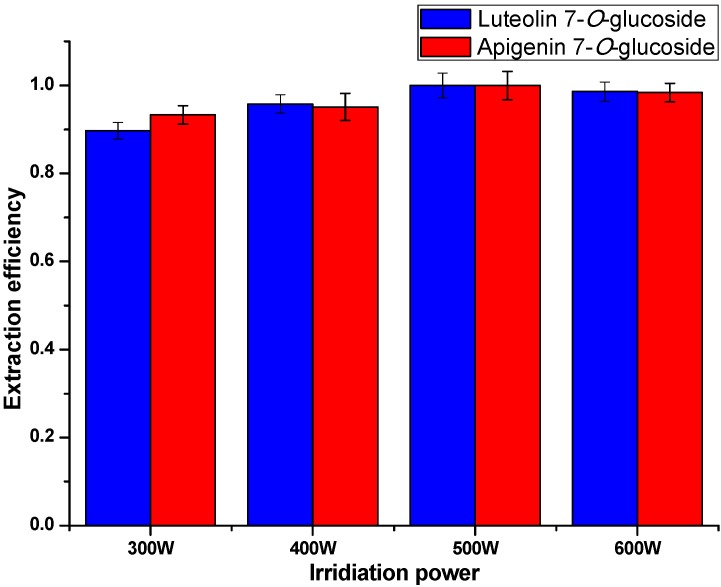
Extraction efficiency under different irradiation powers.

Referring to previous research [28], surfactants must be mobile in solution to form the necessary aggregates. Thermal energy is the driving force for IL-based surfactants to aggregate since it provides the major part of the motion. To examine the effect of irradiation power variance on the relative extraction efficiency, the microwave power was separately set at 300 W, 400 W, 500 W and 600 W. Other conditions including concentration of ILs, extraction time and matrix-to-solvent ratio were applied using the results optimized above. As shown in Figure 8, the irradiation power did not show a significant effect. 500 W was selected as a suitable condition.

### 2.4. Optimization of Parameters by Response Surface Method (RSM)

The univariate method for optimization of the extraction process has limited ability in evaluating the interactions of the extraction conditions. Therefore, to achieve the high extraction efficiency and pick out the most significant conditional parameters, all the possible factors including the concentration of ionic liquid (A), extraction time (B), microwave power (C) and the matrix-to-solvent ratio (D) were comprehensively optimized by the response surface method (RSM). Luteolin 7-*O*-glucoside and apigenin 7-*O*-glucoside were chosen as the indicating compounds. The RSM analysis could model the relationship between the response (extraction efficiency) and the conditions.

For luteolin 7-*O*-glucoside, there is only a 0.01% chance that a “Model F-Value” this large could occur due to noise. Values of “Prob > F” less than 0.0500 indicate the model terms are significant. In this case A, C, D, AB, CD, A^2^, C^2^, D^2^ are all significant model terms. The “Pred R-Squared” of 0.8801 is in reasonable agreement with the “Adj R-Squared” of 0.9584. “Adeq Precision” measures the signal to noise ratio. A ratio greater than 4 is desirable. Our ratio of 25.033 indicates an adequate signal. This model can be used to navigate the design space. The final equation in terms of actual factors is:

(1) $$Efficiency=1.174+1.078\times A+5.196\times 10^{-4}\times C-30.856\times D-0.037\times A\times B+5.806\times 10^{-3}\times C\times D-1.459\times A^{2}-6.875\times 10^{-7}\times C^{2}+366.528\times D^{2}$$

For apigenin 7-*O*-glucoside, there is only a 0.01% chance that a “Model F-Value” this large could occur due to noise. Values of “Prob > F” less than 0.0500 indicate the model terms are significant. In this case A, B, D, AB, A^2^, D^2^ are the significant model terms. Values greater than 0.1000 indicate model terms are not significant. The “Pred R-Squared” of 0.7972 is in reasonable agreement with the “Adj R-Squared” of 0.9296. “Adeq Precision” measures the signal to noise ratio. A ratio greater than 4 is desirable. Our ratio of 20.003 indicates an adequate signal. This model can be used to navigate the design space. The final equation in terms of actual factors is:

(2) $$Efficiency=0.525+1.383\times A+0.018\times B+9.373\times D-0.057\times A\times B-1.824\times A^{2}-178.071\times D^{2}$$

**Figure 9:**
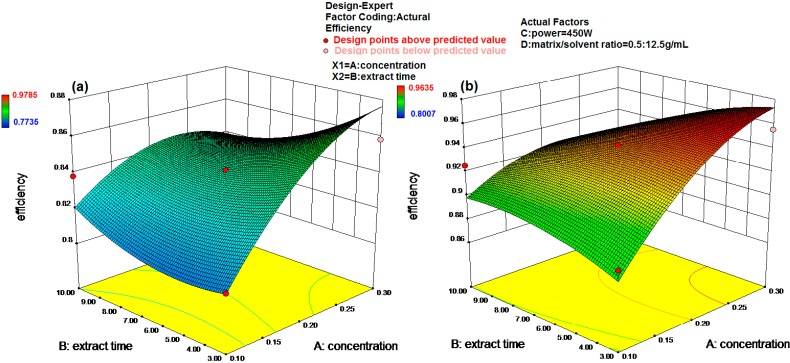
Response surface plots showing the effects of variables on extraction efficiency of two indicator compounds. (**a**) Luteolin 7-*O*-glucoside; (**b**) Apigenin 7-*O*-glucoside.

In both equations, the interaction coefficients for C and D were 450 W and 0.04 (0.5/12.5) g/mL which were optimized by the *Design-Expert* software, so we only need to study the effects of the interactions between factor A and factor B on the extraction efficiency. The response surfaces for the effect of independent variables on extraction efficiency of luteolin 7-*O*-glucoside and apigenin 7-*O*-glucoside are shown in Figure 9. Among all the solutions, when the A = 0.2 M, B = 3 min, C = 600 W, D = 0.5/12.5 g/mL, the extraction efficiency of luteolin7-*O*-glucoside can reach 1; when the A = 0.1 M, B = 6.5 min, C = 450 W, D = 0.5/25 g/mL, the extraction of apigenin 7-*O*-glucoside can reach 1. 

The verification tests were performed five times under the conditions optimized above,* i.e.*, 0.5 g of flower sample was mixed with 12.5 mL of [C_12_mim]Br aqueous solution (0.2 M), and then the suspensions were irradiated for 3 min at an irradiation power of 600 W; 0.5 g of flower sample was mixed with 25 mL of [C_12_mim]Br aqueous solution (0.1 M), and then the suspensions were irradiated with irradiation power 6.5 min at 450 W; Based on the optimized result by single factor (0.25 M, 4 min, 0.5/20 g/mL, 500 W), the actual extraction efficiencies on average were 1.05 (luteolin 7-*O*-glucoside) and 1.02 (apigenin 7-*O*-glucoside), with errors of about 5% and 2%, respectively.

### 2.5. Morphological Changes of Flowers after Extraction

**Figure 10:**
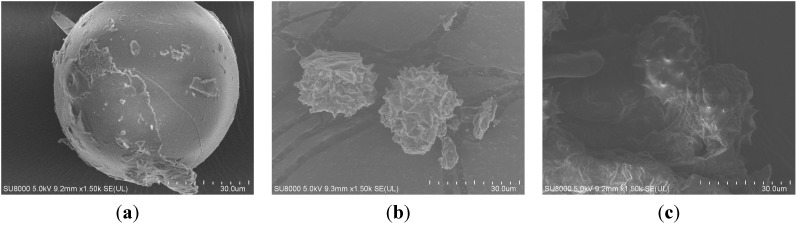
Scanning electron micrographs (30.0 μm, 5.0 kV) of *Chrysanthemum morifolium* Ramat flowers. (**a**) Untreated sample; (**b**) After heating extraction by ethanol 2 h; (**c**) After IL-MWE.

In order to observe the morphological changes before and after the extraction for the mechanism study, the flower material samples were examined by scanning electron microscopy. The scanning electron micrographs of *Chrysanthemum morifolium* Ramat flowers after traditional heating extraction (2 h by ethanol) and microwave-assisted extraction (4 min by [C_12_mim]Br aqueous solution) are shown in Figure 10. The traditional treatment shrank the cells. On the contrary, the microwave-assisted extraction made the cells more withered and even ruptured. This could be ascribed to the special effect of microwaves whereby the solvent permeated into the cells of the sample matrixes under the higher temperatures and then extracted the target compounds into the micelles.

## 3. Experimental Section

### 3.1. Reagents and Materials

Chromatographic grade methanol and formic acid were purchased from J & K Chemical Ltd. (Shanghai, China). All ionic liquids were synthesized and fully characterized using previously published methods [51]. Analytically pure reagents including bromoethane, 1-bromobutane, 1-bromohexane, 1-bromooctane, 1-bromodecane, 1-bromododecane, 1-bromotatradecane, 1-bromohexadecane and *N*-methylimidazole were bought from Aladdin (Seattle, WA, USA) and used without further purification. All the other reagents obtained from Beijing Chemical Reagents Co. (Beijing, China) were of analytical grade. Deionized water was purified by a Milli-Q water purification system from Millipore (Bedford, MA, USA). The flowers of *Chrysanthemum morifolium* Ramat were purchased from a local drug store, in Hangzhou, China. All samples were dried, triturated, passed through an 80 mesh sieve, and stored in closed desiccators at 4 °C until the next extraction. A same batch of samples was used throughout this study to be representative of variable hardness and density.

### 3.2. Instrumentation

A household microwave-assisted extraction unit (Glanz, Shunde, China) equipped with a 2450-MHz magnetron and modified with a water condenser was used for extraction of the target phytochemicals from Chinese herbs. This equipment was run at atmospheric pressure with a maximum power of 700 W. Chromatographic analysis was performed on an Agilent 1100 series HPLC system (Agilent Technologies, Santa Clara, CA, USA) including a G1311AQuat-Pump, a G1322 degasser, a G1314A variable-wavelength detector, a model 7725i injection valve with a 20-μL loop, and an Agilent Zorbax Extend column (150 mm × 4.6 mm I.D., 5 μm, 120 Å) in a column temperature controller. Instrument control and data analysis were carried out by Agilent Chromatographic ChemStation. ^1^H and ^13^C-NMR spectra were obtained with an Advance 500 NMR spectrometer (Bruker, Billerica, MA, USA).

### 3.3. 1-Alkyl-3-methylimidazolium-type ILs

The synthesis of eight 1-alkyl-3-methylimidazolium bromide ILs including 1-ethyl-3-methylimidazolium ([C_2_mim]Br), 1-butyl-3-methylimidazolium ([C_4_mim]Br), 1-hexyl-3-methyl-imidazolium ([C_6_mim]Br), 1-octyl-3-methylimidazolium ([C_8_mim]Br), 1-decyl-3-methylimidazolium ([C_10_mim]Br), 1-dodecyl-3-methylimidazolium ([C_12_mim]Br), 1-tetradecyl-3-methylimidazolium ([C_14_mim]Br), and 1-hexadecyl-3-methylimidazolium ([C_16_mim]Br) was based on literature procedures [53]. A mixture of 1-alkylbromide (60 mmol) and 1-methylimidazole (50 mmol) was placed in above microwave-assisted reactor. Upon continuous stirring and intermittent microwave irradiation for 30 s plus several additional 15 s bursts at 240 W, the ionic liquid started to form. The whole formation of ionic liquid could be monitored visibly as it turned from clear solution to opaque and finally clear again. The product, a yellow viscous liquid, was washed with diethyl ether (3 × 10 mL) followed by hexane (3 × 10 mL), and dried under vacuum at 80 °C for 24 h to give a product yield of 90%. The purity of ILs was checked by their ^1^H-NMR and ^13^C-NMR spectra. The characterization by NMR spectroscopy showed that there were no organic impurities in the ILs. The structures of the ILs are listed in Table 2.

**Table 2:** The chemical structures and CMCs of the eight ILs.

ILs	Cations	Anions	CMC (mol/L)
[C_2_mim]Br	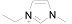	Br^–^	2.5 ± 0.5
[C_4_mim]Br	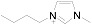	Br^–^	0.8 ± 0.1
[C_6_mim]Br	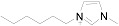	Br^–^	0.6 ± 0.2
[C_8_mim]Br	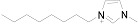	Br^–^	0.15 ± 0.05
[C_10_mim]Br	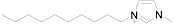	Br^–^	0.03 ± 0.01
[C_12_mim]Br	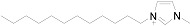	Br^–^	0.0109
[C_14_mim]Br	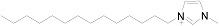	Br^–^	0.0028
[C_16_mim]Br	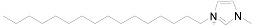	Br^–^	0.0005

CMC: critical micelle concentration; obtained from references [54,55,56].

### 3.4. IL-Based Microwave-Assisted Extraction

Firstly, flower sample (0.5 g) was mixed with different IL solutions (10 mL), and then the suspensions were irradiated in the above microwave-assisted extraction system with running cold water and continuous mechanical stirring. The effects of extraction time, matrix-to-solvent ratio, irradiation power, IL concentration and alkyl chain length on the relative extraction efficiency were systematically investigated. After each irradiation, the obtained extracts were cooled to room temperature and diluted to 50 mL with deionized water for further HPLC analysis. A blank control extraction trial was also carried out with ethanol and water as extraction solvents.

### 3.5. Conventional Reference Extraction Method

Heat reflux extraction was selected as the reference method for extraction of the flavonoid glycosides. A water-bath was performed with 0.5 g sample and 10 mL 95% ethanol (which was optimized as the best extractant) in a 100 mL flask and the suspensions were allowed to boil for 2 h. After extraction, the obtained extracts were cooled to 25 °C, and then diluted to 50 mL with deionized water for subsequent HPLC analysis.

### 3.6. HPLC-MS^n^ Analysis 

The LC operating parameters, including mobile phase, elution program, flow rate, column temperature, a monitoring wavelength were set as follows [5,52]: the mobile phase consisted of acetonitrile (A) and 1% formic acid aqueous solution (B). Gradient elution was programmed as 0–40 min, 10%–26% A, gradient to 60% at 50 min, turned to 95% in last 5 min and held several minutes. The diluted extracts were directly injected into the high performance liquid chromatography at a flow rate of 1.0 mL/min under 35 °C. The ultraviolet detector was set at 350 nm. Under these conditions, the ten flavonoid glycosides were baseline-separated. The component identification was carried out by comparison of their characteristic mass spectra with literature data.

## 4. Conclusions

This study demonstrated that the IL-based surfactant solutions showed excellent ability to selectively extract 10 flavonoid glycosides from *Chrysanthemum morifolium* Ramat when assisted by microwave irradiation. [C_12_mim]Br was identified as the best extraction medium. The optimized extraction conditions based on different methods are listed as follows: 0.25 M, 4 min, 0.5/20 g/mL, 500 W, by single factor; 0.2 M, 3 min, 0.5/12.5 g/mL, 600 W, by RSM and referred to luteolin 7-*O*-glucoside; 0.1 M, 6.5 min, 0.5/25 g/mL, 450 W, by RSM and referred to apigenin 7-*O*-glucoside. This study indicated that the solution with the addition of an appropriate ionic liquid as an extraction medium using microwave-assistance is a rapid and effective method to extract flavonoid glycosides from plants. 

## Abbreviations

CID: collision induced dissociation
IL: ionic liquid
MAE: microwave-assisted extraction
MS^n^: multi-stage mass spectrometry analysis
UAE: ultrasonic-assisted extraction
A: concentration of ionic liquid
TCM: traditional Chinese medicine
B: extraction time
RSM: response surface method
C: microwave power
D: matrix-to-solvent ratio

## References

[1] Tsuji-Naito K., Saeki H., Hamano M. (2009). Inhibitory effects of *Chrysanthemum* species extracts on formation of advanced glycation end products. Food Chem..

[2] Xie Y., Qu J., Wang Q., Wang Y., Yoshikawa M., Yuan D. (2012). Comparative Evaluation of Cultivars of *Chrysanthemum morifolium* Flowers by HPLC-DAD-ESI/MS Analysis and Antiallergic Assay. J. Agric. Food Chem..

[3] Ukiya M., Akihisa T., Yasukawa K., Kasahara Y., Kimura Y. (2001). Constituents of Compositae Plants. 2. Triterpene Diols, Triols, and Their 3-*O*-Fatty Acid Esters from Edible *Chrysanthemum* Flower Extract and Their Anti-inflammatory Effects. J. Agric. Food Chem..

[4] Clifford M.N., Wu W., Kirkpatrick J., Kuhnert N. (2007). Profiling the Chlorogenic Acids and Other Caffeic Acid Derivatives of Herbal Chrysanthemum by LC-MS^n^. J. Agric. Food Chem..

[5] Lai J.P., Lim Y.H., Su J., Shen H.M., Ong C.N. (2007). Identification and characterization of major flavonoids and caffeoylquinic acids in three *Compositae* plants by LC/DAD-APCI/MS. J. Chromatogr. B. Anal. Technol. Biomed. Life Sci..

[6] Zhang H., Kang M., Zhang H., Yu Y. (2014). Matrix solid phase dispersion extraction for determination of flavonoids in the flower of *Chrysanthemum morifolium* Ramat. by capillary zone electrophoresis. Anal. Methods.

[7] Liang F., Hu C., He Z., Pan Y. (2014). Anarabinogalactan from flowers of *Chrysanthemum morifolium*: structural and bioactivity studies. Carbohydr. Res..

[8] Zheng Y., Wang X.S., Fang J. (2006). Two acidic polysaccharides from the flowers of *Chrysanthemum morifolium*. J. Asian Nat. Prod. Res..

[9] Kim H.J., Lee Y.S. (2005). Identification of new Dicaffeoylquinic acid from *Chrysanthemum morifolium* and their antioxidant activities. Planta Med..

[10] Chung H.S. (2006). Phenolic Compounds with Antioxidant Activity on DPPH Free Radical Scavenging and Inhibition of Xanthine/Xanthine Oxidase from the Flowers of *Chrysanthemum morifolium*. J. Food Sci. Nutr..

[11] Xie Y.Y., Yuan D., Yang J.Y., Wang L.H., Wu C.F. (2009). Cytotoxic activity of flavonoids from the flowers of *Chrysanthemum morifolium*on human colon cancer Colon205 cells. J. Asian Nat. Prod. Res..

[12] Harborne J.B., Williams C.A. (2000). Advances in flavonoid research since 1992. Phytochemistry.

[13] Altagirone S.C., Ossi C.R., Oggi A.P., Anelletti F.O.R., Atali P.G.I.N., Runetti M.B., Iello F.B.A., Iantelli M.P. (2000). Flavonoids apigenin and quecetin inhibit melanoma growth and metastatic potential. Int. J. Cancer.

[14] Lee J.S., Kim H.J., Lee Y.S. (2003). A new anti-HIV flavonoid glucuronide from *Chrysanthemum morifolium*. Planta Med..

[15] Mason T.J., Lorimer J.P. (2002). Applied Sonochemistry. The Uses of Power Ultrasoundin Chemistry and Processing.

[16] Li H., Chen B., Yao S. (2005). Application of ultrasonic technique for extracting chloro-genic acid from *Eucommia ulmodies* Oliv. (*E. ulmodies*). Ultrason. Sonochem..

[17] Lu Y., Ma W., Hu R., Dai X., Pan Y. (2008). Ionic liquid-based microwave-assisted extraction of phenolic alkaloids from themedicinal plant *Nelumbonucifera* Gaertn. J. Chromatogr. A.

[18] Du F.Y., Xiao X.H., Li G.K. (2007). Application of ionic liquids in the microwave-assisted extraction of *trans*-resveratrol from *Rhizma Polygoni Cuspidati*. J. Chromatogr. A.

[19] Shen J., Shao X. (2005). A comparison of accelerated solvent extraction, Soxhlet extraction, and ultrasonic-assisted extraction for analysisof terpenoids and sterols in tobacco. Anal. Bioanal. Chem..

[20] Hen J.S., Zhixiu X.U., Ai J.C., Hao X.S. (2006). Determination of pyrethroid residues in tobacco by means of solid phase microextration and GC/MS with the aid of ultrasonic assisted extraction using water as extracting solvent. Anal. Sci..

[21] Quezada N., Asencio M., Delvalle J.M., Aguilera J.M., Gomez B. (2004). Antioxidant Activity of Crude Extract, Alkaloid Fraction, and Flavonoid Fraction from Boldo (*Peumus boldus* Molina) Leaves. Food Chem. Toxicol..

[22] Zhang Y., Liu C., Qi Y., Li S., Pan Y., Li Y. (2015). Circulating ultrasound-assisted extraction, countercurrent chromatography, and liquid chromatography for the simultaneous extraction, isolation, and analysis of the constituents of *Uncaria tomentosa*. J. Chromatogr. A.

[23] Anna F.D., Musumarra G., Noto R. (2014). A multivariate insight into ionic liquids toxicities. RSC Adv..

[24] Jastorff B., Störmann R., Ranke J., Mölter K., Stock F. (2003). How hazardous are ionic liquids? Structure–activity relationships and biological testing as important elements for sustainability evaluation. Green Chem..

[25] Romero A., Santos A., Tojo J., Rodr A. (2008). Toxicity and biodegradability of imidazolium ionic liquids. J. Hazard Mater..

[26] Ghavre M., Byrne O., Altes L., Surolia P.K., Spulak M., Quilty B., Thampi K.R., Gathergood N. (2014). Low toxicity functionalised imidazolium salts for task specific ionic liquid electrolytes in dye-sensitised solar cells: A step towards less hazardous energy production. Green Chem..

[27] Latała A., Marcin N., Stepnowski P. (2010). Toxicity of imidazolium ionic liquids towards algae. Influence of salinity variations. Green Chem..

[28] Van Rantwijk F., Sheldon R.A. (2007). Biocatalysis in Ionic Liquids. Chem. Rev..

[29] Plechkova N.V., Seddon K.R. (2008). Applications of ionic liquids in the chemical industry. Chem. Soc. Rev..

[30] Passos H., Freire M.G., Coutinho J.A.P. (2014). Ionic liquid solutions as extractive solvents for value-added compounds from biomass. Green Chem..

[31] Liu T., Sui X., Zhang R., Yang L., Zu Y., Zhang L. (2011). Application of ionic liquids based microwave-assisted simultaneous extraction of carnosic acid, rosmarinic acid and essential oil from *Rosmarinus officinalis*. J. Chromatogr. A.

[32] Ma C., Liu T., Yang L., Zu Y., Chen X., Zhang L. (2011). Ionic liquid-based microwave-assisted extraction of essential oil and biphenyl cyclooctene lignans from *Schisandra chinensis* Baill fruits. J. Chromatogr. A.

[33] Zhang L., Hu S., Chen X., Bai X., Li Q. (2013). A new ionic liquid–water–organic solvent three phase microextraction for simultaneous preconcentration flavonoids and anthraquinones from traditional Chinese prescription. J. Pharm. Biomed. Anal..

[34] Chen F., Mo K., Liu Z., Yang F., Hou K., Li S., Zu Y., Yang L. (2014). Ionic Liquid-Based Vacuum Microwave-Assisted Extraction Followed by Macroporous Resin Enrichment for the Separation of the Three Glycosides Salicin, Hyperin and Rutin from *Populus* Bark. Molecules.

[35] Liu Z., Jia J., Chen F., Yang F., Zu Y., Yang L. (2014). Development of an Ionic Liquid-Based Microwave-Assisted Method for the Extraction and Determination of Taxifolin in Different Parts of *Larix gmelinii*. Molecules.

[36] Ma C., Liu T., Yang L., Zu Y., Wang S., Zhang R. (2011). Study on ionic liquid-based ultrasonic-assisted extraction of biphenyl cyclooctenelignans from the fruit of *Schisandra chinensis* Baill. Anal. Chem. Acta.

[37] Pino V., Anderson J.L., Ayala J.H., González V., Afonso A.M. (2008). The ionic liquid 1-hexadecyl-3-methylimidazolium bromide as novel extracting system for polycyclic aromatic hydrocarbons contained in sediments using focused microwave-assisted extraction. J. Chromatogr. A.

[38] Ressmann A.K., Zirbs R., Pressler M., Gaertner P., Bica K. (2013). Surface-active Ionic Liquids for Micellar Extraction of Piperine from Black Pepper. Z. Naturforsch..

[39] Vicente F.A., Malpiedi L.P., e Silva F.A., Pessoa A., Coutinho J.A.P., Ventura S.P.M. (2014). Design of novel aqueous micellar two-phase systems using ionic liquids as co-surfactants for the selective extraction of (bio)molecules. Sep. Purif. Technol..

[40] Passos H., Trindade M.P., Vaz T.S.M., da Costa L.P., Freire M.G., Coutinho J.A.P. (2013). The impact of self-aggregation on the extraction of biomolecules in ionic-liquid-based aqueous two-phase systems. Sep. Purif. Technol..

[41] Wang P., Ma C., Chen S., Zhu S., Lou Z. (2014). Ionic Liquid-Based Ultrasonic/Microwave-Assisted Extraction of Steroidal Saponins from *Dioscorea zingiberensis* C.H.Wright. Trop. J. Pharm. Res..

[42] Li X.J., Yu H.M., Gao C., Zu Y.G., Wang W., Luo M., Gu C.B., Zhao C.J., Fu Y.J. (2012). Application of ionic liquid-based surfactantsin the microwave-assisted extraction for the determination of four main phloroglucinols from *Dryopteris fragrans*. J. Sep. Sci..

[43] Zhang D.Y., Yao X.H., Duan M.H., Luo M., Wang W., Fu Y.J., Zu Y.G., Efferth T. (2013). An effective negative pressure cavitation-microwaveassisted extraction for determination of phenolic compounds in *P. calliantha* H. Andr.. Analyst.

[44] Xu W., Chu K., Li H., Zhang Y., Zheng H., Chen R., Chen L. (2012). Ionic Liquid-Based Microwave-assisted Extraction of Flavonoids from *Bauhinia championii (Benth.) Benth*. Molecules.

[45] Wei W., Fu Y., Zu Y., Wang W., Luo M., Zhao C., Li C., Zhang L., Wei Z. (2012). Ionic liquid-based microwave-assisted extraction for the determination of flavonoids glycosides in pigeon pea leaves by high performance liquid chromatography-diodearray detector with pentafluorophenyl column. J. Sep. Sci..

[46] Ballesteros-Gómez A., Sicilia M.D., Rubio S. (2010). Supramolecular solvents in the extraction of organic compounds. A review. Anal. Chem. Acta.

[47] Cuyckens F., Claeys M. (2004). Mass spectrometry in the structural analysis of flavonoids. J. Mass Spectrom..

[48] Cuyckens F., Claeys M. (2005). Determination of the glycosylation site in flavonoid mono-*O*-glycosides by collision-induced dissociation of electrospray-generated deprotonated and sodiated molecules. J. Mass Spectrom..

[49] Lin L.Z., Harnly J.M. (2010). Identification of the phenolic components of chrysanthemum flower (*Chrysanthemum morifolium* Ramat). Food Chem..

[50] Bowers J., Butts C.P., Martin P.J., Vergara-gutierrez M.C., Heenan R.K. (2004). Aggregation Behavior of Aqueous Solutions of Ionic Liquids. Langmuir.

[51] Blesic M., Marques M.H., Plechkova N.V., Seddon K.R., Rebelo L.P.N., Lopes A. (2007). Self-aggregation of ionic liquids: Micelle formation in aqueous solution. Green Chem..

[52] Zeng H., Wang Y., Kong J., Nie C., Yuan Y. (2010). Ionic liquid-based microwave-assisted extraction of rutin from Chinese medicinal plants. Talanta.

[53] Varma R.S., Namboodiri V.V. (2001). An expeditious solvent-free route to ionic liquids using microwaves. Chem. Commun..

[54] Goodchild I., Collier L., Millar S.L., Prokes I., Lord J.C.D., Butts C.P., Bowers J., Webster J.R.P., Heenan R.K. (2007). Structural studies of the phase, aggregation and surface behavior of 1-alkyl-3-methylimidazolium halide + water mixtures. J. Colloid Interface Sci..

[55] Dong B., Li N., Zheng L.Q., Yu L., Inoue T. (2007). Surface Adsorption and Micelle Formation of Surface Active Ionic Liquids in Aqueous Solution. Langmuir.

[56] Dong B., Zhao X.Y., Zheng L.Q., Zhang J., Li N., Inoue T. (2008). Aggregation behavior of long-chain imidazolium ionic liquids in aqueous solution: Micellization and characterization of micelle microenvironment. Colloids Surf. A Physicochem. Eng. Asp..

